# When It's Not a Sexually Transmitted Infection (STI): A Diagnostic Challenge of Severe Lipschütz Ulcer in a Young Woman

**DOI:** 10.7759/cureus.96781

**Published:** 2025-11-13

**Authors:** Ashmi Bhattacharya, Alex Rowland, Sahruda Gandham, Nicola Roberts

**Affiliations:** 1 Department of Obstetrics and Gynaecology, Bedford Hospital, Bedfordshire Hospitals NHS Foundation Trust, Bedford, GBR

**Keywords:** acute ulceration, genital ulcers, gynaecological emergency, herpes simplex, lipschütz ulcer, non-sexually transmitted, painful vulvar ulcer

## Abstract

Genital ulcers are often attributed to sexually transmitted infections (STIs), yet a notable subset originates from non-venereal causes. Misdiagnosis can lead to unnecessary treatments, social stigma, and emotional distress. Lipschütz ulcer is a relatively uncommon, non-sexually transmitted condition characterized by the sudden appearance of painful, necrotic ulcers in the vulvar region. It predominantly affects adolescents and young women and is frequently underrecognized. Acute genital ulcers have been linked to infections such as Epstein-Barr virus (EBV) and other viral or bacterial pathogens, including SARS-CoV-2, and, rarely, can occur following SARS-CoV-2 vaccination, though the exact cause often remains unclear.

We present the case of a 21-year-old woman who developed severe vulvar ulceration and swelling over seven days, leading to urinary retention. While abroad, she was initially misdiagnosed with herpetic ulcers, but a brief prodromal febrile illness preceded lesion onset. Examination revealed bilateral "kissing" ulcers on the labia majora with irregular borders and a fibrinoid necrotic base. Careful evaluation ruled out infectious, inflammatory, and traumatic causes, including necrotizing fasciitis, infected herpetic lesions, and sexual assault. Correlating her clinical features led to the diagnosis of Lipschütz ulcer. Supportive management with corticosteroids resulted in rapid symptom relief and complete recovery.

This case emphasizes the importance of considering Lipschütz ulcer in young women presenting with sudden, painful genital ulcers, particularly following a febrile illness and in the absence of sexual exposure. Raising awareness among gynaecologists, general practitioners, dermatologists, and paediatricians can reduce misdiagnosis, prevent unnecessary interventions, and alleviate patient distress. Sharing such cases helps improve recognition and supports the development of standardized diagnostic and management strategies for this underdiagnosed condition.

## Introduction

Genital ulcers often raise the suspicion of sexually transmitted infections (STIs). However, it is essential to adopt a broader differential diagnosis that includes non-STI causes, many of which are actually more prevalent. Misdiagnosis as a venereal disease can lead to improper treatment and significant psychological distress.

Lipschütz ulcer (LU), also referred to as acute genital ulcers or acquired genital ulceration, is a rare, self-limiting condition predominantly affecting adolescent and young women, typically under the age of 20 years [1,2]. First described by Benjamin Lipschütz in 1912 as "ulcus vulvae acutum Lipschütz", this entity is characterized by the sudden onset of painful, necrotic genital ulcers following a prodromal phase of nonspecific systemic symptoms such as fever, malaise, myalgia, and lymphadenopathy [2,3].

The ulcers most frequently involve the labia minora but may also appear on the labia majora, vestibule, fourchette, or introitus [1,3,4]. Lesions can present as single or multiple, with sharply demarcated, raised borders, and are often covered with a greyish exudate or necrotic eschar. Bilateral "kissing lesions" are considered a characteristic finding [4,5]. The condition typically resolves spontaneously within 2-6 weeks, and recurrence is uncommon [5].

Although the precise etiology of LU remains unclear, several infectious agents have been implicated, including Epstein-Barr virus (EBV), cytomegalovirus (CMV), influenza A and B, parvovirus B19, and adenovirus. More recently, cases associated with SARS-CoV-2 infection or vaccination have been reported [6]. LU remains a diagnosis of exclusion, as it may clinically resemble a wide range of infectious (e.g., syphilis, herpes genitalis, chancroid), inflammatory (e.g., Behçet disease, Crohn's disease), and traumatic causes, including sexual assault [7,8].

Given its rarity and nonspecific presentation, LU is often underdiagnosed and underreported in clinical practice. This article presents a case of LU in a 21-year-old woman, highlighting its clinical features, diagnostic approach, and differential diagnoses. Increasing awareness of this condition among clinicians may facilitate timely recognition, reduce unnecessary investigations and treatments, and improve patient outcomes.

## Case presentation

A 21-year-old woman presented to the emergency department with a one-week history of severe vulvar pain, genital ulceration, and progressive vulvar swelling, resulting in acute urinary retention due to pain. The patient reported that while on holiday abroad, she awoke to notice what appeared to be a purplish bruise on both labia majora, more prominent on the left side. Over the following days, the lesion evolved into a painful ulcer on the left labia majora with a necrotic margin, greyish exudate, and a yellow, non-fetid discharge. Smaller reciprocal "kissing ulcers" were also noted on the right labia majora.

She initially sought medical attention overseas, where she was provisionally diagnosed with genital herpes complicated by secondary bacterial infection. Pelvic ultrasonography performed at that time was unremarkable. She received a course of oral acyclovir and doxycycline, but her symptoms did not improve.

Upon presentation to our emergency department, her pain and swelling were severe enough to cause urinary retention, necessitating catheterization. She reported a recent episode of a "severe cold" approximately one week prior to ulcer onset, which had confined her to bed for a day. She denied any previous history of genital herpes or other STIs. She reported being in a stable monogamous relationship and had not been sexually active for three months prior to symptom onset. A sensitive discussion excluded any history of sexual trauma or assault.

**Figure 1:**
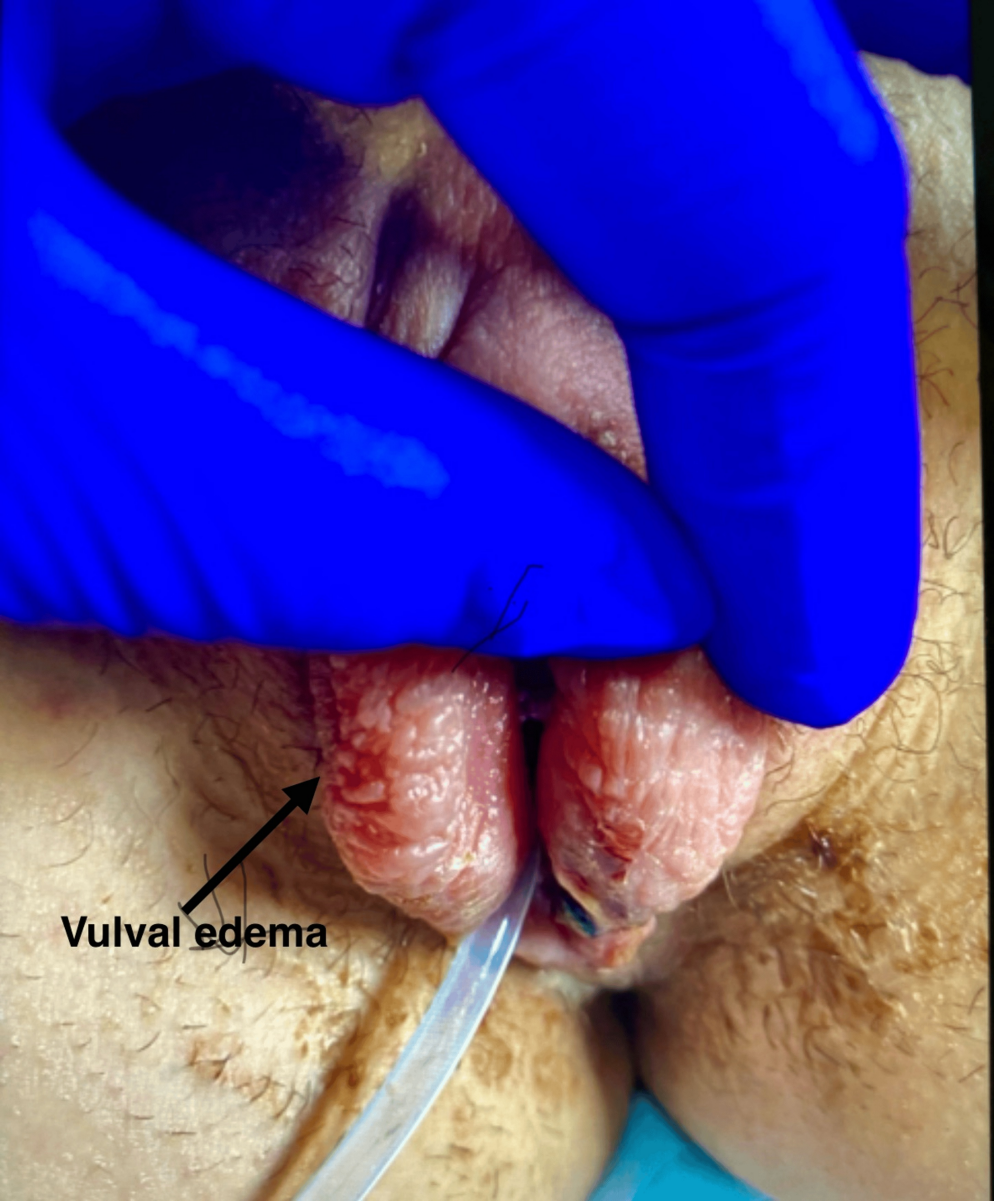
Vulval edema at presentation Severe vulval edema at presentation in a case of Lipschütz ulcer, necessitating urinary catheterization to relieve urinary retention caused by marked labial swelling and pain.

Her medical history was significant for coeliac disease, with no prior surgeries. There was no history of oral ulcers or similar lesions elsewhere on the body. On examination, she was haemodynamically stable. Inspection of the genital area revealed marked swelling and induration of both the labia minora and majora, accounting for her dysuria and retention (Figure 1).

**Figure 2:**
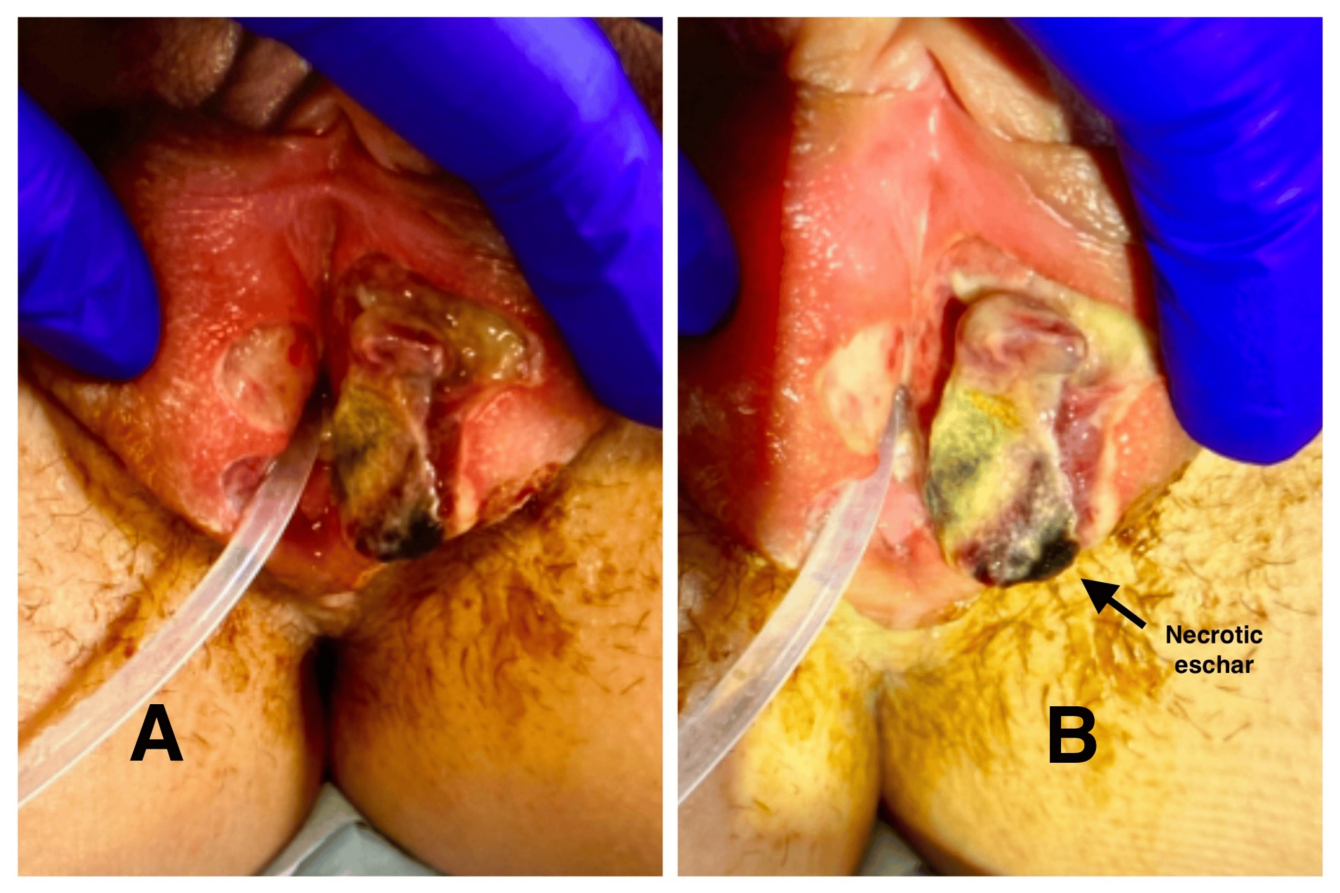
(A-B) Left labia majora ulcer with necrotic base (A) A 2.5×1.5 cm ulcer involving the left labia majora, extending toward the vaginal introitus. The lesion shows irregular, sharply demarcated borders with an erythematous halo and a fibrinoid base. (B) Closer view of the ulcer demonstrating greyish black necrotic eschar overlying the fibrinoid base.

On gentle separation of the labia majora, a 2.5×1.5 cm ulcer was observed involving the left labia majora, extending toward the vaginal introitus. The ulcer exhibited irregular, sharply demarcated borders, an erythematous halo, and a fibrinoid base with greyish necrotic material (Figure 2A-2B).

**Figure 3:**
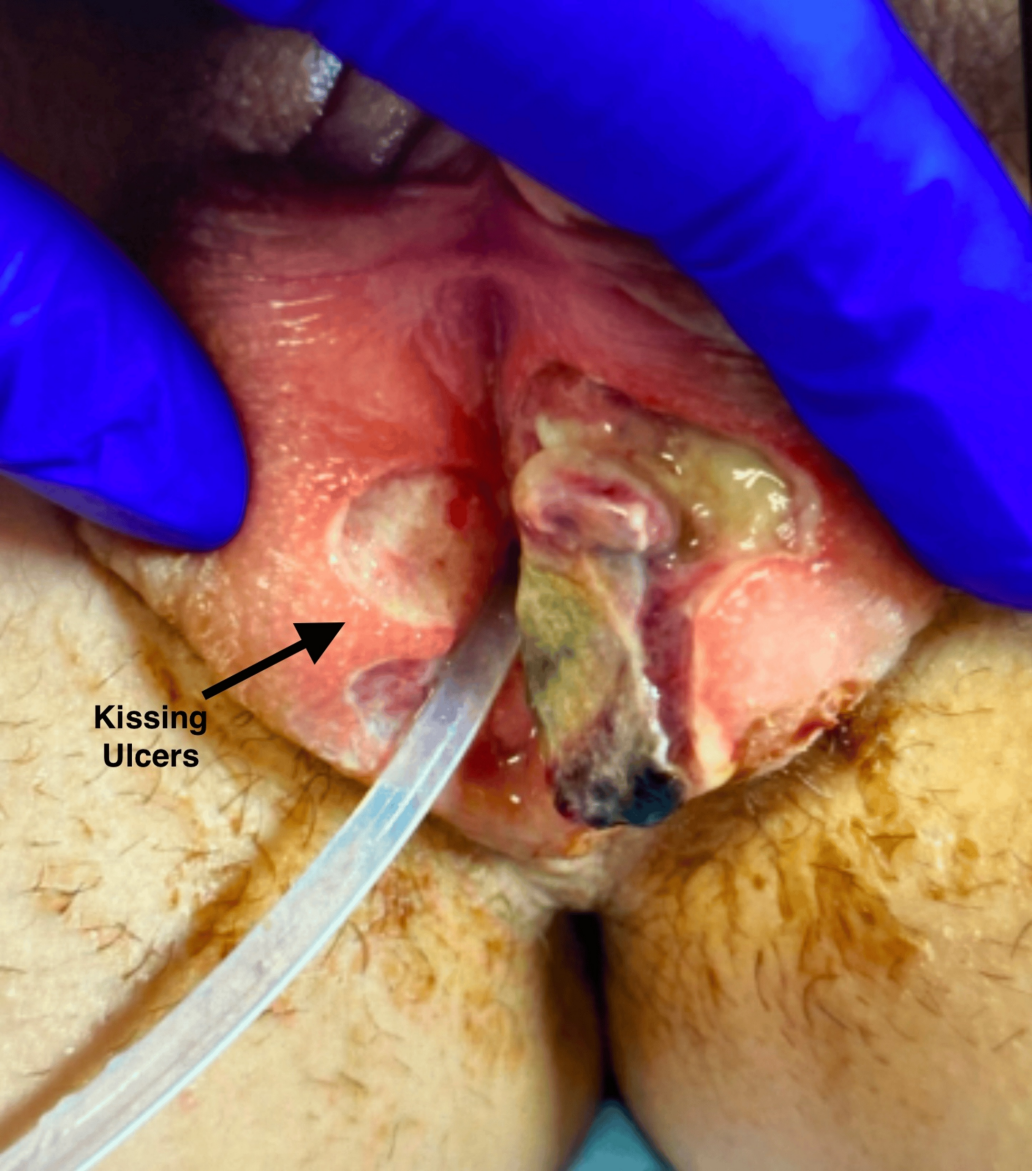
Contralateral kissing ulcer on the right labia

A contralateral ulcer of similar appearance was present on the right labia majora, producing the characteristic "kissing lesion" pattern. The ulcers were exquisitely tender on palpation, and no inguinal lymphadenopathy was noted (Figure 3).

Baseline laboratory investigations, including complete blood count and serum biochemistry, were within normal limits. Throat and genital swabs yielded no bacterial or fungal growth. Serological testing for hepatotropic viruses (hepatitis B surface antigen (HBsAg), anti-hepatitis C virus (anti-HCV) antibody, anti-hepatitis A virus (anti-HAV) antibody, immunoglobulin M (IgM)/immunoglobulin G (IgG)) was negative. Screening for *Treponema pallidum *(*Treponema pallidum* hemagglutination assay (TPHA) and venereal disease research laboratory (VDRL) test) was non-reactive, and an oropharyngeal SARS-CoV-2 swab tested negative.

Acute-phase reactants showed mildly elevated C-reactive protein (9.2 mg/L) and a normal white blood cell count (7.2×10⁹/L). Urinalysis and urine culture were unremarkable. Polymerase chain reaction (PCR) testing for herpes simplex virus types 1 and 2 returned negative results. Additional bacterial and sexually transmitted infection cultures from the ulcer were negative, effectively excluding syphilis, chancroid, and human immunodeficiency virus (HIV) infection.

Given the severity and necrotic appearance of the ulcers, the patient was initially managed with analgesia and intravenous flucloxacillin to treat possible secondary bacterial infection. Microbiology was consulted to exclude necrotizing fasciitis and to advise on appropriate antimicrobial therapy. Broad-spectrum intravenous meropenem and clindamycin were commenced as per their recommendation.

Following exclusion of infectious and inflammatory causes, the clinical picture was consistent with a LU, later confirmed with multidisciplinary input from dermatology. Systemic corticosteroid therapy with oral prednisolone 40 mg once daily was initiated, along with continued analgesia and completion of the antibiotic course to minimize the risk of secondary bacterial infection.

The patient showed marked clinical improvement within three days, with resolution of pain and reduction in vulvar edema. She was discharged home after three days on oral antibiotics, a tapering course of prednisolone, and supportive care. Over the following 10 days, her ulcers healed completely with minimal discomfort. At her three-month follow-up, she reported full recovery with complete epithelialization of the lesions and no residual scarring.

## Discussion

LU, or acute genital ulcer, is a clinical diagnosis characterized by the abrupt onset of painful genital ulcers, typically in sexually inactive adolescent females [9]. These ulcers usually begin with a prodromal stage marked by viral-like symptoms such as fever, fatigue, and general malaise, followed by the appearance of one or more vulvar ulcers measuring between 0.1 cm and 2.5 cm in diameter [6].

LUs can present in three clinical variants based on their course and morphology. Table 1 summarizes the three clinical variants of LU, outlining their characteristic morphology and typical clinical course [10-12].

**Table 1:** The three clinical variants of Lipschütz ulcer with descriptions of their characteristic morphology and typical clinical course Table adapted from Moise et al. [10], Torok et al. [11], and Lai et al. [12].

Variant	Morphology	Clinical course
Gangrenous	Hyperacute, deep ulcers with a white-grey base	The most common form, often associated with systemic symptoms such as fever and malaise
Miliary	Fibrinous, superficial, purulent, small ulcers (~1 cm in diameter)	Generally not associated with systemic symptoms; heals completely without scarring or recurrence
Chronic or relapsing	Recurrent ulcers of variable depth and extent	Characterized by repeated episodes over time, with possible residual scarring

In this case report, we describe the gangrenous type of LU. Regarding prevalence, the occurrence of LU ranges between 4% and 35% among women presenting with acute genital ulcers [2]. Although the true prevalence is uncertain, LU may account for up to 30% of acute vulvar ulcerations and is often underrecognized or misdiagnosed [13]. These ulcer predominantly affects adolescents and young women (most cases ≤20 years), though some studies suggest a higher incidence in children and older women [1,2,14].

The etiopathogenesis of LU is unknown. Several case reports and series have identified associations with positive serology for various viral and bacterial pathogens, including EBV, CMV, and *Mycoplasma pneumoniae*. More recently, links have been reported with SARS-CoV-2 infection and, in some cases, following COVID-19 vaccination [4]. Two main pathophysiological mechanisms have been proposed: the first postulates that direct cytotoxic effects of viral infection lead to mucocutaneous ulceration accompanied by systemic febrile symptoms; the second suggests that immune complex formation during the acute phase of infection may trigger a type III hypersensitivity reaction. In this process, immune complexes activate the complement cascade, inducing inflammation that results in microthrombosis and subsequent tissue necrosis. Additional hypotheses include hematogenous dissemination or local autoinoculation as contributing mechanisms [2-4].

When evaluating a potential case of LU, it is essential to recognize that this condition represents a diagnosis of exclusion. Comprehensive clinical assessment and targeted investigations are required to rule out infectious and non-infectious causes of genital ulceration. Typically, the ulcers are deep, ranging from 0.1 to 2.5 cm in diameter, with sharply demarcated erythematous borders and a necrotic base covered by grey exudate or grey-black eschar. They are often accompanied by labial edema, fever, and regional lymphadenopathy and may be preceded by nonspecific systemic symptoms. The presence of bilateral, symmetrical "kissing lesions" is also a distinctive diagnostic clue [4]. In our patient, these characteristic clinical findings were observed.

According to the diagnostic framework proposed by Sadoghi et al., both major and minor criteria should be assessed [14]. Table 2 enumerates the major and minor diagnostic criteria for LU.

**Table 2:** Major and minor diagnostic criteria for Lipschütz ulcer Table adapted from Sadoghi et al. [14].

Diagnostic criteria for Lipschütz ulcer
Major criteria
Acute onset of at least one painful vulval ulcer
Exclusion of infectious and non-infectious etiologies
Minor criteria
Localization of the ulcer at the vestibule or labia minora
History of abstention from sexual activity within the preceding three months or absence of prior sexual activity
Presence of flu-like symptoms
Occurrence of a systemic infection within 2-4 weeks prior to ulcer onset

A definitive diagnosis requires fulfillment of both major criteria and at least one minor criterion [14].

The differential diagnosis of genital ulcers encompasses a broad range of infective and non-infective etiologies [8]. Among STIs, common causes include herpes simplex virus, *Treponema pallidum* (syphilis), and HIV. Non-STI infectious causes include EBV, *Mycoplasma pneumoniae*, CMV, *Toxoplasma gondii*, influenza virus, mumps virus, *Salmonella *species, parvovirus B19, *Mycobacterium tuberculosis*, *Leishmania*, and *Entamoeba histolytica* [4,8].

Non-infective causes are diverse and include drug reactions, immunobullous diseases, aphthosis, Behçet's disease, inflammatory bowel disease, erosive variants of lichen planus and lichen sclerosus et atrophicus, premalignant and malignant lesions, pyoderma gangrenosum, and hidradenitis suppurativa. It is also critical to consider and rule out sexual abuse when evaluating genital ulcers, particularly in younger patients. Certain systemic conditions can be distinguished by their characteristic features: in Crohn's disease, genital ulcers tend to be recurrent and are typically located in the perianal region, accompanied by gastrointestinal manifestations. In Behçet's syndrome, a history of recurrent oral and genital aphthae is common, often associated with uveitis and retinal vasculitis [4,5,8].

After systematically excluding all possible infective and non-infective causes based on the patient's clinical history, physical examination, and laboratory investigations, including bacteriological, fungal, and viral assays, and by applying the diagnostic criteria, our case was found to fulfill both major criteria and three minor criteria, confirming the diagnosis of LU.

LUs are typically self-limiting, with spontaneous recovery occurring within approximately three weeks. Management primarily focuses on symptomatic relief and promoting ulcer healing. Reported treatments include analgesics, topical corticosteroids, and antibiotics, while a short course of systemic corticosteroids (prednisolone 0.5 mg/kg for one to two weeks) may be beneficial in cases of severe pain, multiple ulcers, or necrotic lesions. Complete healing generally occurs within six weeks without scarring, with an average healing time reported between 16 and 21 days (range 5-52 days) [8].

Given the potential association of LUs with autoimmune conditions like Sjögren's syndrome, regular follow-up, weekly until healing and annually thereafter, is recommended to monitor for systemic diseases such as inflammatory bowel disease or Behçet's disease [3,4]. In our case, telephone follow-up at three and eight weeks revealed the complete resolution of symptoms with no residual complaints.

Most of the previously reported case reports describe LU in adolescent, often virgin patients aged 10-13 years and associate it with viral illnesses such as COVID-19 or other systemic viral infections [4,6-8]. In contrast, our patient was a 21-year-old woman who had been sexually inactive for three months and developed severe ulcers following a prodromal viral-like illness; however, due to the one-week delay before presentation, the specific viral trigger could not be identified.

## Conclusions

LU is an uncommon but important and often underrecognized cause of acute genital ulceration. It is extremely painful and often distressing for patients. Awareness among dermatologists, paediatricians, general practitioners, and gynaecologists is vital to avoid misdiagnosis and unnecessary procedures. The condition is not sexually transmitted and usually heals spontaneously. Diagnosis is clinical and made by exclusion after ruling out infections, autoimmune diseases, trauma, and other causes. Early recognition allows the timely use of corticosteroids and effective pain relief, reducing patient anxiety. In children, the ulcers can be mistaken for signs of sexual abuse, leading to unnecessary investigations and family distress. Because formal guidelines are lacking and reports remain limited, further research is needed to understand the etiology and to establish clear diagnostic and treatment protocols.
